# Overdosage of PPD immunotherapy causing tuberculosis-like skin lesions^⋆^^☆^

**DOI:** 10.1016/j.abd.2023.07.007

**Published:** 2024-04-20

**Authors:** John Verrinder Veasey, Victória Cerqueira Elia, Ana Estela Ribeiro, Rute Facchini Lellis

**Affiliations:** a Dermatology Clinic, Hospital da Santa Casa de São Paulo, São Paulo, SP, Brazil; b Faculdade de Ciências Médicas, Santa Casa de São Paulo, São Paulo, SP, Brazil

Dear Editor,

The present report describes a 60-year-old patient who, concerned about the COVID-19 pandemic, underwent immunotherapy with purified protein derived from *M. bovis* (PPD), a substance used for the Mantoux tuberculin skin test. The performed therapy, under medical indication, consisted of subcutaneous self-applications, once a day, on three consecutive days, in the abdominal region. Within a week, the patient developed intense inflammation at the injection sites characterized by painful erythematous nodules, with the release of purulent secretion (Fig. 1), followed by spontaneous improvement and cicatricial lesions within a month.

**Fig. 1:**
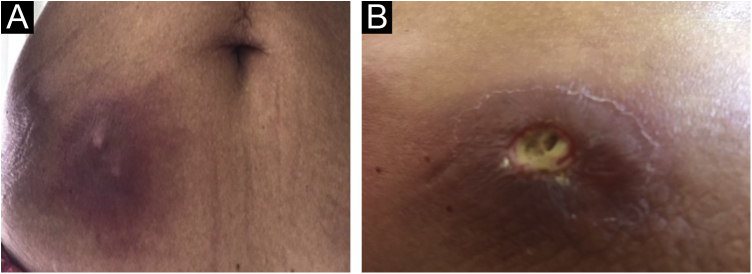
(A and B) Abscesses at PPD inoculation sites, one week after the procedure.

After one year, she sought dermatological help aiming at aesthetically improving these lesions. On clinical examination, she had three subcutaneous plaques that were more palpable than visible, measuring approximately three centimeters each, overlaid by atrophic skin, with the elimination of caseous secretion upon expression (Fig. 2). There were no systemic symptoms, cough, or other skin lesions. She brought the leaflet for the injected product,^1^ and with the hypothesis of a PPD-overdose reaction, a skin biopsy was performed, and the histological hematoxylin & eosin (HE) stained slides showed the tuberculoid-type granulomatous dermatosis with caseous necrosis, a condition compatible with “PPD-itis” (Fig. 3). The caseous material was sent for PCR analysis using the Xpert MTB/RIF® method as reported in the literature,^2^ with a negative result for *M. tuberculosis*. Other tests such as complete blood count, IGRA, and chest X-ray were unremarkable, indicating localized reactive disease, restricted to the inoculation sites.

**Fig. 2:**
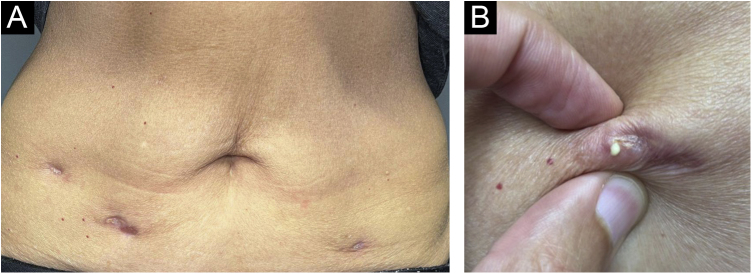
(A and B) Clinical appearance one year after the injections, with nodular lesions that released caseous material upon expression.

**Fig. 3:**
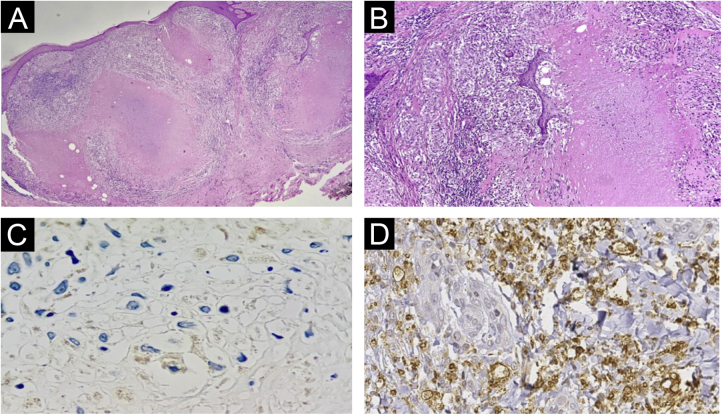
Histopathology of lesions of “PPD-itis”. (A) (Hematoxylin & eosin, ×40). Showing granulomatous reaction with extensive caseous necrosis. (B) (Hematoxylin & eosin, ×100). Detail of the tuberculoid granuloma with caseous necrosis. (C and D) Negative immunohistochemistry for *Mycobacterium tuberculosis*.

Treatment with surgical excision of the lesions was chosen, and the material was sent for culture for micobacteria which was negative, and new histopathological analysis, negative immunohistochemistry for *M. tuberculosis*, Ziehl-Neelsen and Fite Faraco stains, corroborating the diagnosis of “PPD-itis.” The patient progressed with complete healing of the surgical wounds, maintaining follow-up in the Dermatology and Infectious Diseases Outpatient Clinics (Fig. 4).

**Fig. 4:**
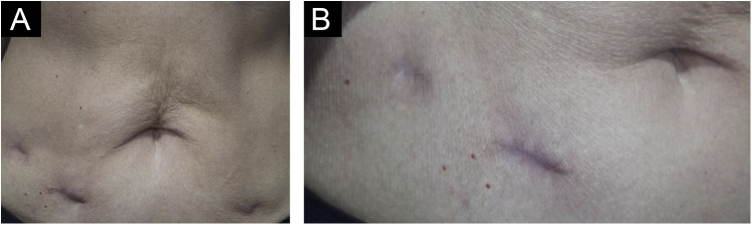
(A and B) Clinical appearance after surgical excision of the lesions (two months postoperatively).

Cutaneous tuberculosis (CTB) is a rare form of micobacteriosis, representing 1% to 2% of all forms of extrapulmonary tuberculosis. The main etiological agent is *Mycobacterium tuberculosis* and, occasionally, *Mycobacterium bovis* and bacillus Calmette-Guérin (BCG vaccine), an attenuated strain of *M. bovis*.^3^ Several studies support the use of the BCG vaccine as immunotherapy in the treatment of neoplasms (bladder tumors and melanoma), asthma, Parkinson's disease, recurrent respiratory papillomatosis and even recurrent verruca vulgaris,^4^, ^5^, ^6^ with rarer descriptions of the use of PPD with the same purpose.^7^

During the COVID-19 pandemic, an association of less severe courses of the disease was observed in patients previously vaccinated with the BCG vaccine,^8^ leading some physicians to recommend immunotherapy with PPD, as occurred with the patient in the present report. It is important to highlight that PPD is part of daily medical practice in countries with a high prevalence of tuberculosis, such as Brazil, and that cases of adverse reactions are rare when the test is adequately carried out.

The leaflet of the product injected by the patient highlights that the substance is indicated for immunological research of delayed sensitivity (cellular immunity) to the bacterium *Mycobacterium tuberculosis* and it is not indicated for use as immunotherapy. It also expounds on the risks of overdose, which can occur in conditions similar to the one observed in the present case.^1^ If the authors had not had access to the leaflet of the product used by the patient, the condition might have been misdiagnosed as CTB due to BCG injection, instead of a reactive granulomatous dermatosis due to PPD overdose.

The histopathological examination provided important information. The Ziehl-Neelsen and Fite Faraco stains did not show any evidence of the agent, reinforcing the fact that it was a reactive dermatosis without the presence of the micobacteria. The negative immunohistochemical reaction indicates the absence of parasite antigens in the reaction site. Therefore, the lesion is related to the presence of the injected product, precisely a purified protein derived from micobacteria. The negative PCR test of the caseous material reinforces this reasoning. Finally, the presence of granulomas with central caseous necrosis in the H&E stained slides demonstrates that this pattern so frequently reported in infections caused by tuberculous micobacteria is related to the immunological stimulus caused by the protein antigen, not necessarily to the bacteria.

The proposed treatment with surgical excision was indicated after an interdisciplinary discussion with the Infectious Disease team. Described as a therapeutic option for refractory CTB lesions,^3^ it has shown to be a good alternative for the localized cutaneous condition and has brought resolution without exposing the patient to prolonged drug treatment, which could bring undesirable side effects, especially considering her age group. The patient has been followed for six months, with no signs of recurrence.

## Financial support

None declared.

## Authors' contributions

John Veasey: Design and planning of the study; data collection, or analysis and interpretation of data; drafting and editing of the manuscript or critical review of important intellectual content; collection, analysis, and interpretation of data; effective participation in research orientation; intellectual participation in the propaedeutic and/or therapeutic conduct of the studied cases; critical review of the literature; approval of the final version of the manuscript.

Victoria Elia: Data collection, or analysis and interpretation of data; drafting and editing of the manuscript or critical review of important intellectual content; collection, analysis and interpretation of data; intellectual participation in the propaedeutic and/or therapeutic conduct of the studied cases; critical review of the literature; approval of the final version of the manuscript.

Ana Ribeiro: Intellectual participation in the propaedeutic and/or therapeutic conduct of the studied cases.

Rute Lellis: Data collection, or analysis and interpretation of data; drafting and editing of the manuscript or critical review of important intellectual content; collection, analysis and interpretation of data.

## Conflicts of interest

None declared.
